# Blood glucose and insulin levels in patients with peripheral vestibular disease

**DOI:** 10.1016/S1808-8694(15)30521-8

**Published:** 2015-10-18

**Authors:** Ana Paula Serra, Karen de Carvalho Lopes, Ricardo S. Dorigueto, Fernando Freitas Ganança

**Affiliations:** 1 Specialist in Neurotology - UNIFESP-EPM, Otolaryngologist; 2 Specialist in Neurotology - UNIFESP-EPM, Otolaryngologist. MSc student -UNIFESP-EPM; 3 Student in Sciences - UNIFESP-EPM, Otolaryngologist; 4 Medicine - UNIFESP/EPM, Adjunct Professor, Head of the Outpatient Ward and Department of Vestibular Rehabilitation Sector - Neurotology Discipline - UNIFESP/EPM. Professor-Collaborator - Graduate Program in Vestibular Rehabilitation and Social Inclusion - UNIBAN

**Keywords:** glucose, insulin, carbohydrate metabolism, dizziness, vertigo

## Abstract

Metabolic disorders can cause dizziness.

**Aim:**

to study the prevalence of glucose and glucose-insulin alterations in patients with peripheral vestibular disorders by studying the four-hour glucose-insulin curve; to check at what time there was the highest prevalence of altered cases and whether the glucose and insulin curves together are better than the isolate glucose curve and fasting glucose curve.

**Materials and Methods:**

retrospective study, analyzing 81 four-hour glucose-insulin curves in patients with peripheral vestibular dizziness.

**Results:**

Four-hour glucose-insulin curve alterations happened in 87.7% of the patients. Hypoglycemia was seen in 61.7% of the cases, hyperinsulinemia in 55.5%, hyperglycemia in 27.2%, glucose intolerance in 12.3% and hypoinsulinemia in 1.2%. Normal tests were seen in 12.3 % of the cases and altered fasting glucose in 23.5%.

**Conclusions:**

The four-hour glucose-insulin curve analysis showed that 87.7% of the patients with dizziness and suspicion of peripheral vestibular disorder had glucose or insulin metabolism disorders. The highest number of alterations was seen up to the third and fourth hour of the glucose-insulin curve. The glucose and insulin curves together overcame the glucose curve alone and fasting glucose curve in regards of the prevalence of altered cases.

## INTRODUCTION

Dizziness is considered the second most prevalent symptom before 65 years of age, and it is the most frequent after such age. About 85% of patients with dizziness have some sort of vestibular disorder^1^.

Benign Paroxysmal Positional Vertigo, Endolymphatic hydrops and metabolic-origin vertigo are the most relevant among the most frequent vestibular disorders in our country^2^, ^3^, ^4^, ^5^. The latter can account for 17.1% of vestibular disorders^1^.

Numerous metabolic disorders impact inner ear functioning^2^, ^3^, ^4^, ^5^, ^6^. Disorders involving the metabolism of carbohydrates represent frequent causes of vestibular and auditory alterations, and in most of the cases they stem from glucose metabolism disorders^1^, ^5^, ^7^, ^8^. Patients usually complain of vertigo, a feeling of floating, tinnitus, weakness, sweat and tremor^8^. Hypoglycemia, hyperglycemia and even mild alterations in insulin levels are already enough to cause changes which impact the labyrinth^8^, ^9^, ^10^, ^11^. Metabolic disorders may act as the major etiological factor in vestibular dysfunctions or be a worsening factor in a pre-existing vestibular disorder.

Fasting glucose and glycemic index can provide us information on the presence of hyper or hypoglycemia in the metabolic assessment of a patient with vestibular disorder. The insulin index can also aid in this assessment. Charles and Kirtane^12^, ^13^ found more alterations in the insulin index in patients with vestibular disorders when compared to normal volunteers, with a statistically significant difference.

Nonetheless, there is no consensus whether the glucose-insulin index could have a diagnostic advantage over the glycemic index alone and fasting glucose in patients with vestibular disorders. As far as the glycemic and glucose-insulin indexes are concerned, there is also some doubt regarding test duration, which would have a greater likelihood to check alterations in these patients.

Thus, the goals of this study were to use the four-hour glucose-insulin index to identify the prevalence of glucose and glucose-insulin alterations in patients with diagnostic hypothesis of peripheral vestibular disease, out of the spell period; to check at what point of the exam a greater prevalence of altered cases was found; to check whether the glycemic and insulin indexes together are better than the glycemic index alone and fasting glucose alone as to the prevalence of cases with alterations.

## METHODS

This study was submitted to and approved by the Ethics in Research Committee of the institution where it was carried out, protocol # 0558/08.

This is a retrospective study based on the investigation of the charts of 81 patients complaining of dizziness and diagnostic suspicion of peripheral vestibular syndrome. We included the patients submitted to the interview, ENT physical and neurologic examination, audiometric evaluation, vestibular test and 4-hour glucose-insulin index measuring glycemic and insulin indexes after a 12-hour fasting and 30, 60, 90, 120, 180 and 240 minutes after the ingestion of 75g de dextrose. We excluded those patients with a prior diagnosis of Diabetes Mellitus.

The results from the glycemic index were plotted, analyzed and classified according to the criteria from the American Diabetes Association published in 2008 and the insulin index interpretation was based on the Kraft criteria (1975)^14^, ^15^, ^16^, ^17^.

Fasting glucose values above 99mg/dl and values higher than or equal to 200mg/dl for the glycemic index were considered altered, characterizing the hyperglycemia-type alteration; the cases in which the glucose was higher then or equal to 140mg/dl at the 120 minute-point in the glycemic index were considered as glucose intolerance; having one or more values below 55mg/dl, at any point of the test was classified as hypoglycemia in absolute value; a glucose drop greater than 1mg per minute was considered hypoglycemia in relative value.

The insulin curve was analyzed according with Kraft^15^ criteria, having the following findings as abnormalities: fasting insulin greater then 25μUI/ml, late insulin peak (at 120 or 180 minutes), 120-minute insulin value greater than 50 μUI/dl and the summation of insulin values of the 2nd and 3rd hour greater than 60 μUI/ml, characterizing hyperinsulinemia-type of alteration; cases with all the insulin values lower than 50 μUI/dl were characterized as hypoinsulinemia.

The results obtained through the analysis of the glycemic and glucose-insulin indexes were distributed according with the time of collection and the type of test carried out (fasting glucose, 2-hour glycemic index, 3-hour glucose-insulin index, four-hour glycemic index or four-hour glucose-insulin index), and an abnormal value already made the test altered until its end.

We used the chi-squared test in order to check which test had the greatest number of altered cases and in what duration time. The level of significance adopted was 0.05.

## RESULTS

Glycemic and insulin level analyses showed that during fasting, 23.5% of the glucose values were high, characterizing hyperglycemia. There was no insulin alteration at this time.

Up to the second hour, the glycemic index presented 61.7% of alterations, while the glucose-insulin index was of 79.0%.

**Table 1:** Comparing the prevalence of cases altered in the fasting glucose and two, three and four-hour glycemic and glucose-insulin indexes assessments.

Test	n (%)	p
FG	19 (23,4%)	0,01
GC 2 hours	50 (61,7%)	
FG	19 (23,4%)	0,01
GIC 2 hours	64 (79,0%)	
FG	19 (23,4%)	0,01
GC 3 hours	58 (71,6%)	
FG	19 (23,4%)	0,01
GIC 3 hours	71 (87,7%)	
FG	19 (23,4%)	0,01
GC 4 hours	64 (79,0%)	
FG	19 (23,4%)	0,01
GIC 4 hours	71 (87,7%)	

FG: fasting glucose; GC: glycemic index; GIC: glucose-insulin index.

Up to the third hour, the glycemic index showed 71.6% of alterations, while the glucose-insulin index, 87.7%.

And finally, up to the fourth hour, the glycemic index showed 79.0% of alterations, while the glucose-insulin index had 87.7%.

Among the 4-hour glucose-insulin index we found: 54 patients (66.7%) with hypoglycemia, relative in 35 (64.8%) and in absolute value in 29 (53.7%), 45 subjects (55.5%) with hyperinsulinemia, 22 (27.2%) with hyperglycemia, 10 (12.3%) with glucose intolerance and 1 (1.2%) with hypoinsulinemia, when more than one alteration could be found in the same test.

The two, three and four hour glycemic and glucose-insulin indexes found a greater prevalence of altered cases, with statistically significant differences when compared to two-hour glycemic index and, finally, the three and four-hour glucose-insulin indexes showed the same percentage of altered cases (Table 2).

**Table 2:** Comparison between the prevalence of altered cases in glycemic and glucose-insulin indexes at the different duration periods of these tests.

Test	n (%)	p
GC 2 hours	50 (61,7%)	0,040
GIC 3 hours	71 (87,7%)	
GC 2 hours	50 (61,7%)	0,040
GIC 4 hours	71 (87,7%)	
GIC 3 hours	71 (87,7%)	1,00
GIC 4 hours	71 (87,7%)	

FG: fasting glucose; GC: glycemic index; GIC: glucose-insulin index.

## DISCUSSION

Alterations in carbohydrate metabolism, such as hypoglycemia and hyperglycemia, as well as insulin alterations, represent one of the most frequent causes of balance disorders^1^, ^3^, ^8^, ^18^. The early diagnosis of these disorders is extremely important in the assessment of patients complaining of dizziness^8^, ^11^.

It is known that the labyrinthine structures and, especially the stria vascularis, have a very intense metabolic activity and, therefore, depend on a constant and proper supply of oxygen, glucose and adenosine triphosphate (ATP). Larger energy expenditures are necessary to keep proper concentrations of sodium and potassium in the endolymph, and glucose is fundamental for ATP production within the cells and energy supply for the sodium and potassium pump to work properly.

As such, the metabolic alterations which involve the glucose metabolism can impair energy supply, altering the concentration of ions in the endolymph and perilymph, causing a change in the labyrinthine electric potentials, initiating dizziness^18^, ^19^.

The lack of glucose as energy source for the sodium and potassium pump generates the endolymphatic hydrops caused by the retention of sodium in the endolymphatic space and a consequent water volume increase in such compartment^3^. Murbach found a negative amplitude variation in the distortion product otoacoustic emissions during hypoglycemia and hyperinsulinemia^6^. Mendelson & Roderique^20^ showed a reduction in endocochlear potential and cochlear microphonism during the hypoglycemia phase associated to a reduction in potassium concentration and increase in sodium concentration in the endolymph, proving the sensitiveness of the auditory system associated to variations in endolymph composition.

The dizzy patient assessment, after interview and complete physical exam includes audiologic tests, vestibular function tests, biochemical, biological and hormonal tests, image exams in order to carry out the topographic and etiological diagnosis. The biochemical evaluation suggested by most of the pertaining scientific literature includes fasting glycemia^2^, ^7^, ^9^, ^10^, ^19^ and, eventually, the prolonged glucose-insulin index ^8^, ^15^, ^18^. It is important to stress that the glucose-insulin index provides more information than the simple fasting glucose, such as relative hypoglycemia or in absolute values - delayed insulin peak, hyperinsulinemia and hypoinsulinemia, and the early diagnosis of these alterations and the institution of a proper diet can prevent diabetes and its complications to set in^16^, ^18^, ^21^, ^22^, besides improving neurotological symptoms^8^.

In the present study, in assessing the four-hour glucose-insulin index, the most commonly found alteration was hypoglycemia (66.7%), in accordance with studies from Charles^12^, Carrillo et al.^23^ and Ferreira et al.^24^. Hyperinsulinemia also proved to be an important alteration, occurring in 55.5% of the cases, which was also reported by Mangabeira Albernaz and Fukuda^18^, who found hyperinsulinemia in 86% of the patients with inner ear disorders and clinical suspicion of metabolic etiology, and by D'Avila and Lawinsky^25^ who found hyperinsulinemia in 72% of the cases with Ménière's syndrome investigated by means of the five-hour glucose-insulin index. Ramos^8^ found 38.8% of alterations in the insulin curve when assessing patients with cochlear and/or vestibular syndromes, and normal glycemic curves, in its majority. Mangabeira Albernaz^26^, studied 130 patients with inner ear metabolic disorders and found 83 (63.15%) with hyperglycemia associated with reactive hypoglycemia and hyperinsulinemia.

The prolonged glucose-insulin index has proven to be more efficient in diagnosing carbohydrate metabolism alterations than the fasting glucose alone^1^, ^3^, ^4^, ^8^, ^16^, ^18^. In the present study, when we analyzed the fasting glucose of dizzy patients, we found 23.5% of the cases with altered values, rate which is lower than the rest of the glycemic or glucose-insulin index times, in accordance with ^8^, Fukuda and Mangabeira Albernaz^18^ and Lavinsky et al.^27^, who found a higher rate of glycemic and insulin alterations in the prolonged curves, identifying few fasting glucose alterations.

In the present investigation, we have also noticed that in the glucose-insulin index there was a higher percentage of alterations up to the third and fourth hours of the exam (p<0.001). Nonetheless, there was no diagnostic benefit when we compared the three and four hour curves, both showing 87.7% alterations. Nonetheless, Ferreira et al.^24^, noticed a greater benefit after the third hour of the test, finding hypoglycemia in 50.0% of the cases and always after the third hour.

Glycemic and insulin assessments were more valuable than the glycemic test alone. These data are in agreement with those from Proctor^11^, Mangabeira Albernaz and Fukuda^18^ and Lavinsky et al.^26^ who also found a greater number of alterations in the five-hour glucose-insulin index when compared to the glycemic and insulin indexes alone.

It is worth stressing that in this study, we assessed consecutive patients complaining of dizziness of peripheral vestibular origin, without selecting those with clinical signs and symptoms suggesting metabolic disorder. Thus, glucose and insulin metabolism assessment must not be discarded in all the patients with dizziness, as reported by Caovilla et al.^28^.

## CONCLUSION

The four-hour glucose-insulin index showed 87.7% of patients with dizziness and suspicion of peripheral vestibular dysfunction having glucose and insulin metabolism alterations. The higher number of alterations was found up to the third and fourth hour of the glucose-insulin index and glucose and insulin indexes together were better than the glucose index alone and the fasting glucose on the prevalence of altered cases.
